# Integrating landscape system and meta-ecosystem frameworks to advance the understanding of ecosystem function in heterogeneous landscapes: An analysis on the carbon fluxes in the Northern Highlands Lake District (NHLD) of Wisconsin and Michigan

**DOI:** 10.1371/journal.pone.0192569

**Published:** 2018-02-07

**Authors:** Haile Yang, Jiakuan Chen

**Affiliations:** 1 Institute of Biodiversity Science, Fudan University, Shanghai, People's Republic of China; 2 Department of Ecology and Evolutionary Biology, School of Life Sciences, Fudan University, Shanghai, People's Republic of China; 3 Centre for Watershed Ecology, Institute of Life Science, Nanchang University, Nanchang, Jiangxi, People's Republic of China; University of Oregon, UNITED STATES

## Abstract

The successful integration of ecosystem ecology with landscape ecology would be conducive to understanding how landscapes function. There have been several attempts at this, with two main approaches: (1) an ecosystem-based approach, such as the meta-ecosystem framework and (2) a landscape-based approach, such as the landscape system framework. These two frameworks are currently disconnected. To integrate these two frameworks, we introduce a protocol, and then demonstrate application of the protocol using a case study. The protocol includes four steps: 1) delineating landscape systems; 2) classifying landscape systems; 3) adjusting landscape systems to meta-ecosystems and 4) integrating landscape system and meta-ecosystem frameworks through meta-ecosystems. The case study is the analyzing of the carbon fluxes in the Northern Highlands Lake District (NHLD) of Wisconsin and Michigan using this protocol. The application of this protocol revealed that one could follow this protocol to construct a meta-ecosystem and analyze it using the integrative framework of landscape system and meta-ecosystem frameworks. That is, one could (1) appropriately describe and analyze the spatial heterogeneity of the meta-ecosystem; (2) understand the emergent properties arising from spatial coupling of local ecosystems in the meta-ecosystem. In conclusion, this protocol is a useful approach for integrating the meta-ecosystem framework and the landscape system framework, which advances the describing and analyzing of the spatial heterogeneity and ecosystem function of interconnected ecosystems.

## Introduction

**Fig 1:**
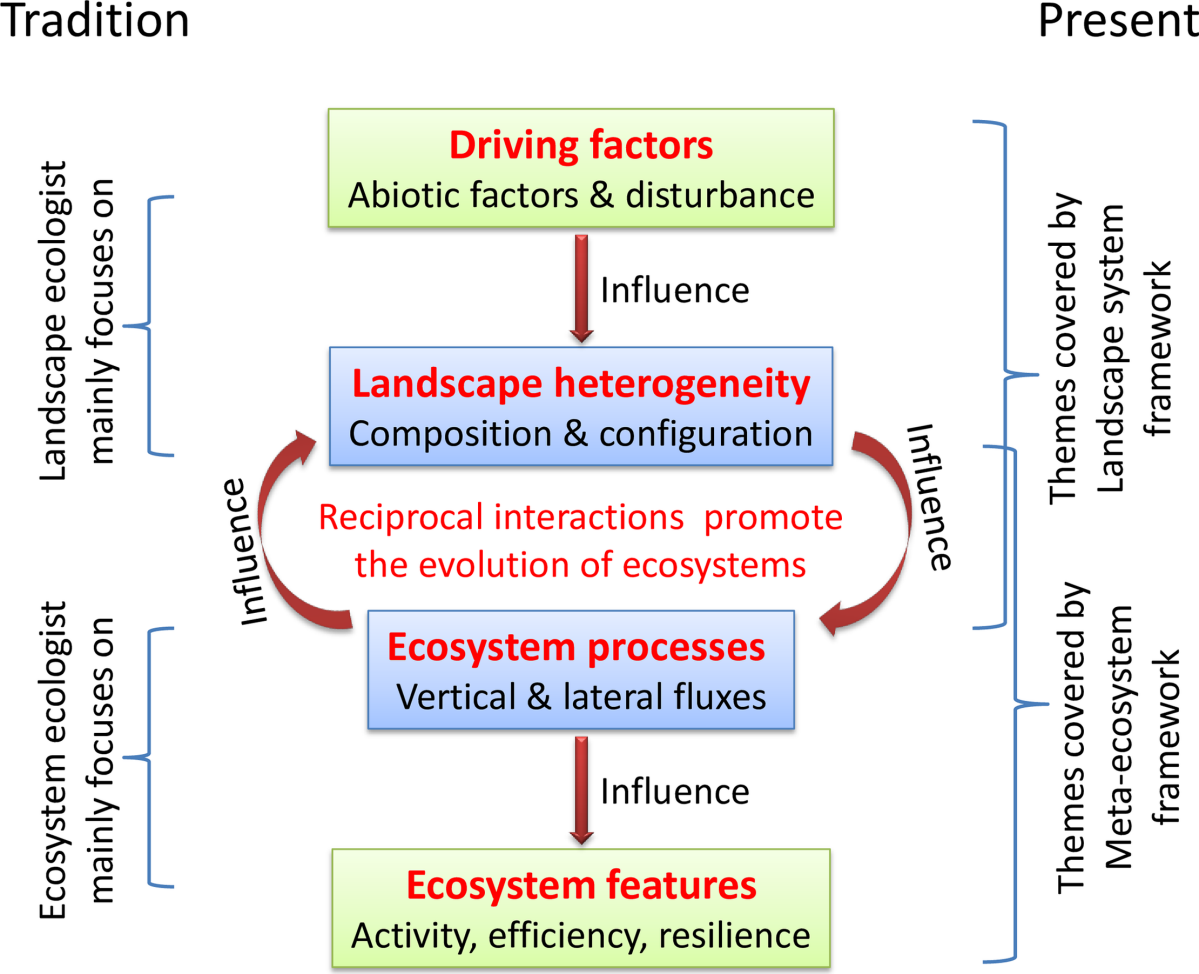
Illustration of what aspects of spatially heterogeneous ecological systems (landscape) do the ecosystem ecology, landscape ecology, meta-ecosystem framework and landscape system framework respectively pay attention to. The primary driving factors of the spatial heterogeneity of ecological systems are abiotic template (includes topography, climate, and substrate) and disturbance (including natural disturbance events, human activities and the long-term legacies of ecosystem evolution). Pattern–process interactions always are reciprocal: ecosystem processes affect landscape patterns, such as nutrient mineralization rates influence plant distributions; landscape patterns affect ecosystem processes, such as the composition and configuration of land use and vegetation cover in a watershed influence nutrient loadings to wetlands, streams and lakes. The ecological flows among ecosystems influence the donor and recipient ecosystems simultaneously, such as the predators feed along streams and carry salmon into riparian forests. Traditionally, landscape ecologists focus on the composition and configuration of the landscape, and ecosystem ecologists focus on the fluxes of matter and energy. Presently, landscape ecologists study ecosystem processes too, but they mainly focus on the causes and consequences of spatial heterogeneity which determines the rates of ecosystem processes (such as net primary productivity and nitrogen mineralization), and how spatial heterogeneity (i.e. land cover composition and configuration) influence ecosystem function (especially, the horizontal movement of water, nutrients and sediments), such as the landscape system framework; ecosystem ecologists study spatial heterogeneity too, but they mainly focus on the effect of spatial flows on the source and target ecosystems (such as the nutrient subsidy and its feedback) and on the higher-level ecosystem (such as the evolution of these interactive ecosystems), such as the meta-ecosystem framework [1]. We need to integrate the landscape system and meta-ecosystem frameworks if we want a much more complete understanding of how spatially heterogeneous ecological systems function.

Ecosystems may be discrete but do not exist in isolation [1]. The set of interactive ecosystems makes up a heterogeneous ecological system (landscape), in which the interactions among ecosystems impact the local features of each ecosystem and the global features of the heterogeneous ecological system. To completely understand and describe its spatial heterogeneity and ecosystem function, we need to integrate ecosystem ecology with landscape ecology [1, 2, 3]. Traditionally, landscape ecologists tended to focus on quantifying the spatial structure of a heterogeneous ecological system [2], in contrast, ecosystem ecology largely considered the ecosystem processes (e.g. fluxes of matter and energy) and functions of an ecological system in the absence of a spatial context [3] (Fig 1). Over the past few decades, several attempts on the integration of ecosystem and landscape ecology have been proposed [1]. These attempts can be divided into two main approaches (Fig 1): (1) the ecosystem-based approach, such as the meta-ecosystem framework [4], and (2) the landscape-based approach, such as the landscape system framework [5]. These two frameworks are complementary, however, incompatible.

To understand the emergent properties arising from the interactions among ecosystems, such as global source–sink constraints, one could use the meta-ecosystem framework proposed by Loreau and others in 2003. A meta-ecosystem is defined as a set of ecosystems connected by flows of energy, materials and organisms across ecosystem boundaries, which is always (*quasi*-) closed or mass-conserving, such as an endorheic watershed in which diverse ecosystems interact with each other according to nutrients cycle [4]. In the meta-ecosystem framework (Table 1), one could use the flows among ecosystems to analyze their impacts on both the source and target local ecosystems [6–8] and how they determine the global features of the meta-ecosystem [9–11].

**Table 1:** Global constraints and holistic properties of a meta-ecosystem [4, 12, 13].

Description	Equation
*Ecosystem dynamic*	$\frac{dX_{i}}{dt}=\sum_{j}F_{ij}-\sum_{j}F_{ji}+G_{i}$
*Global constraints* emerge from spatial fluxes	$\sum_{i,j}F_{ij}-\sum_{i,j}F_{ji}+\sum_{i}G_{i}=0$
*Source-sink constraint within* meta-ecosystems	$\sum_{i}G_{i}=0$
*Size and overall activity* of the meta-ecosystem (TST)	$TST=\sum_{i,j}F_{ij}$
*Organization (constraint)* of meta-ecosystem (AMI)	$AMI=k\sum_{i,j}\frac{T_{ij}}{T}\;\log\left(\frac{T_{ij}T}{T_{i}T_{j}}\right)$
*Ascendency* of meta-ecosystem (A)	$A=\sum_{i,j}T_{ij}\;\log\left(\frac{T_{ij}T}{T_{i}T_{j}}\right)$
*Upper bound of meta-ecosystem ascendency* (C)	$C=\sum_{i,j}T_{ij}\;\log\left(\frac{T_{ij}}{T}\right)$
*Resistance-resilience* of meta-ecosystem (R)	*R* = *C* − *A*

In a (*quasi*-) closed meta-ecosystem, any element that leaves one ecosystem must enter another, and then the global constraints arise from the spatial flows which are constrained by limited organisms, nutrients, energy and information. The holistic properties of meta-ecosystems (TST, AMI, A, C, R) are identified by Ecological Network Analysis (ENA). Total system throughput (TST) is simply the sum of all flows in a meta-ecosystem and reflects the size and overall activity of the meta-ecosystem. Average mutual information (AMI) is an ecological information-based index used to estimate the development or organization of a meta-ecosystem. Ascendency (A) is a key property of a network of flows that quantifies both the level of system activity and the degree of development (organization). Development capacity (C) is the diversity of the system flows scaled by the total system throughput, which serves as an upper bound on system ascendency. Redundancy (R) is the degree to which pathways parallel each other in a network, which can be regarded as resilience, an attribute that is complementary (opposite) to ascendency. In these equations, *X*_*i*_ denotes the size of the organisms, nutrients, energy or information stock in ecosystem *i*, and *G*_*i*_ its local growth rate in the absence of spatial flows among ecosystems, and *F*_*ij*_ the directed spatial flow from ecosystem *i* to ecosystem *j*, *T*_*i*_ = ∑_*j*_*F*_ij_ and *T*_*j*_ = ∑_*i*_*F*_ij_.

**Fig 2:**
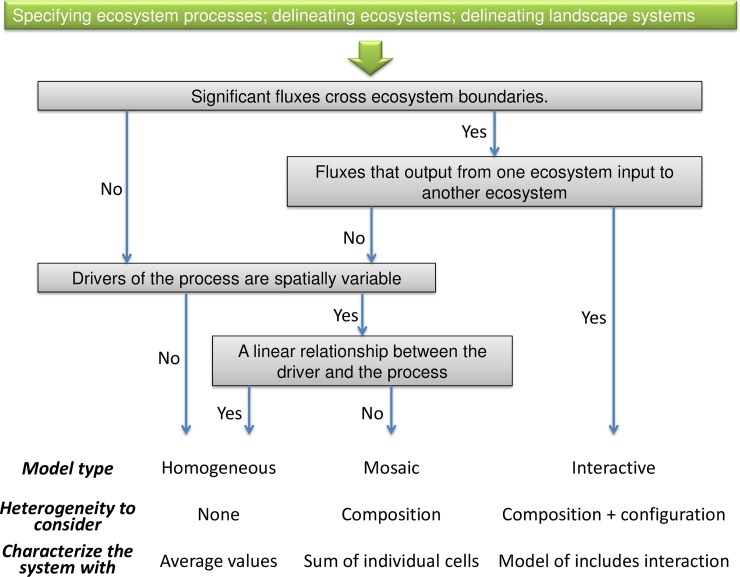
Decision tree for deciding whether spatial features need to be considered in studying ecosystem function in heterogeneous ecological systems (landscapes) (Adapted from fig 24.2 of Lovett and others [5]). The decision tree leads to three different models to dealing with spatial heterogeneity: (*Homogeneous*) assumes spatial homogeneity and characterizes the landscape system by average values of its pools and fluxes; (*Mosaic*) considers composition only using a mosaic approach, in which the behavior of the process in each ecosystem is modeled separately and the results are summed to yield the whole system behavior; and (*Interactive*) considers composition, configuration and interacting ecosystems using an interactive model which incorporates the inter-ecosystem exchanges [5]. That is, perhaps spatial heterogeneity could be safely ignored if there are no lateral fluxes, no spatially variable drivers, and no nonlinearities; however, if there are nonlinearities, then at least, composition of a landscape system must be considered; if lateral fluxes are significant too, then both composition and configuration of a landscape system will be required [1].

To understand the spatial heterogeneity which determines the behavior of a heterogeneous ecological system, such as the spatial pattern of a set of ecosystems connected by drainage system, one could use the landscape system framework proposed by Lovett and others in 2005. A landscape system is the collection of interconnected ecosystems under study, which is always open to inputs and outputs, such as a set of wetlands connected by runoff [5]. In the landscape system framework (Fig 2), one could use the fluxes among relatively homogeneous areas or patches to analyze what aspects of heterogeneity need to be considered and what kind of model (homogeneous, mosaic, or interactive) could be used to appropriately captures the behavior of the system [1, 5]. These models provide frameworks for considering spatial heterogeneity appropriately in studying ecosystem processes and for analyzing driving factors conveniently in studying ecosystem heterogeneity.

The concept of meta-ecosystem is similar to the concept of landscape, but is not equivalent to it. Firstly, a meta-ecosystem is assumed to be (*quasi-*) closed or mass-conserving, while a landscape does not. Secondly, a meta-ecosystem could be spatially disconnected, such as a set of islands connected by seabirds, in contrast, a landscape have to be spatially continuous [4]. So, ecologically, a meta-ecosystem and a landscape are not the same thing. The concept of landscape system is similar to the concept of landscape, but pays more attention on the interactions among its component ecosystems. In another word, the difference between landscape system and landscape is just how we treat the interactions among patches. Ecologically, a landscape system and a landscape are the same thing. Obviously, the meta-ecosystem framework and the landscape system framework are incompatible, although complementary. It would be another step towards the successful integration of ecosystem ecology with landscape ecology if these two frameworks could be integrated (Fig 1). Then we could get an overarching framework for completely understanding a heterogeneous ecological system. In this overarching framework, one could study the driving factors, landscape heterogeneity, ecosystem processes, ecosystem features and the interactions among them systematically (Fig 1).

The objectives of this study are dual: (1) to propose a protocol of integrating the landscape system framework with the meta-ecosystem framework; (2) to demonstrate the application of this protocol using a case study—describing the spatial heterogeneity and ecosystem features of the carbon flows in the Northern Highlands Lake District (NHLD) of Wisconsin and Michigan following this protocol.

## Materials and methods

The protocol of integrating the landscape system framework with the meta-ecosystem framework involved four steps: 1) delineating landscape systems; 2) classifying landscape systems; 3) adjusting landscape systems to meta-ecosystems and 4) integrating landscape system and meta-ecosystem frameworks through meta-ecosystems. As the primary obstacle between these two frameworks is the incompatible difference between the concepts of meta-ecosystem and landscape system, so we integrate these two frameworks through connecting these two concepts.

### Delineating landscape systems

The landscape system—a volume of space that encompasses the ecosystems of interest—is delineated with a defined boundary which distinguishes inputs and outputs from internal circulation [5]. The internal circulation is the ecological flows among ecosystems—relatively homogeneous areas or patches—delineated by defined boundaries. Though frequently depicted on maps as two-dimensional, landscape systems, ecosystems, boundaries and ecological flows are three-dimensional, extending above and below the surface [14, 15].

### Classifying landscape systems

**Fig 3:**
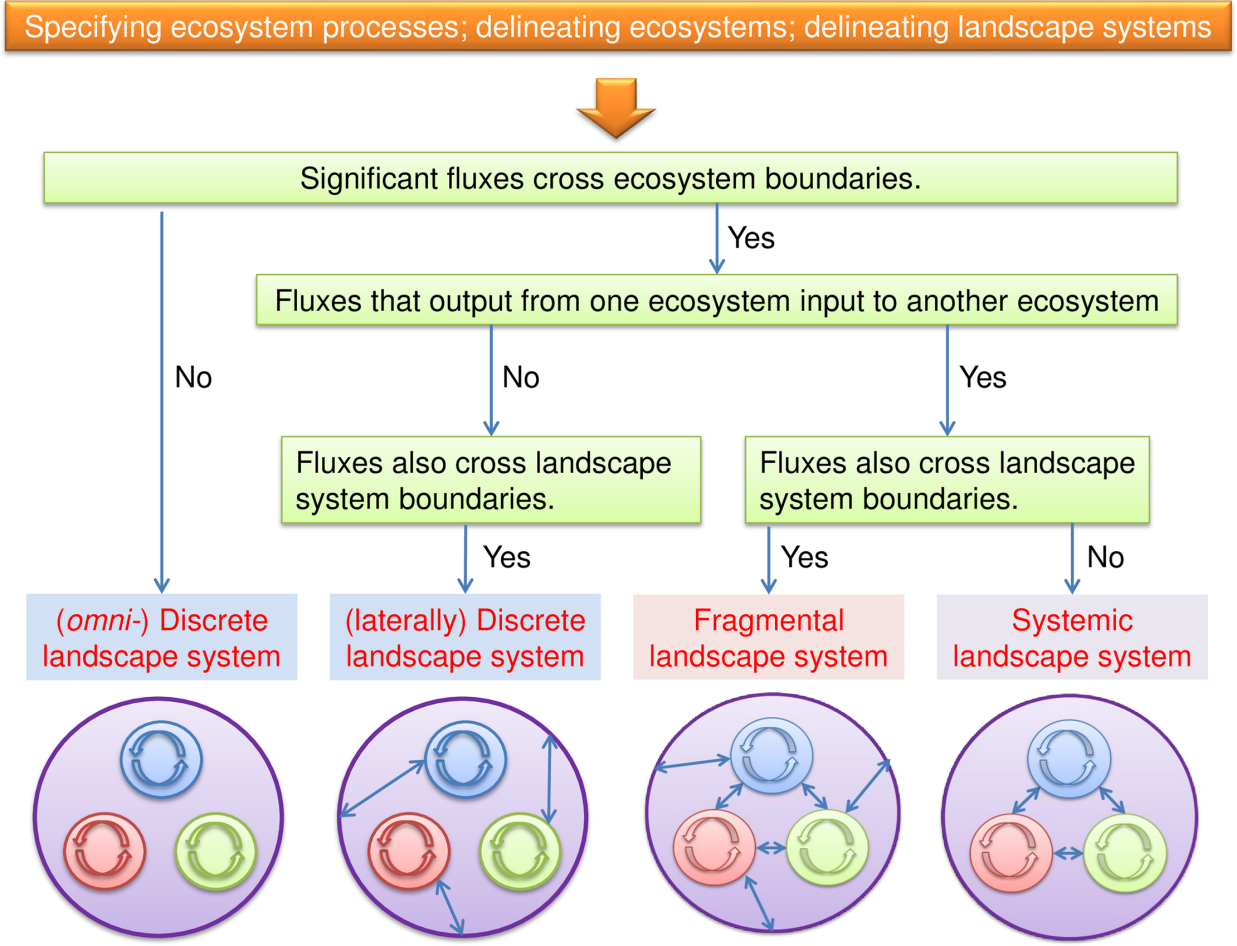
Decision tree of analyzing and classifying landscape systems. The systemic landscape system embraces all significantly interconnected ecosystems; the fragmental one only embraces a part of significantly interconnected ecosystems; the omnidirectionally discrete one embraces a collection of relatively isolated ecosystems; the laterally discrete one embraces a collection of laterally isolated ecosystems. Small circles represent ecosystems; outer circles represent landscape systems; double-sided arrows represent significant fluxes.

Before we can classify the landscape system, we need to specify the ecosystem process(es) of interest. Following that, the decision tree can guide in classifying the landscape system (Fig 3).

### Adjusting landscape systems to meta-ecosystems

**Fig 4:**
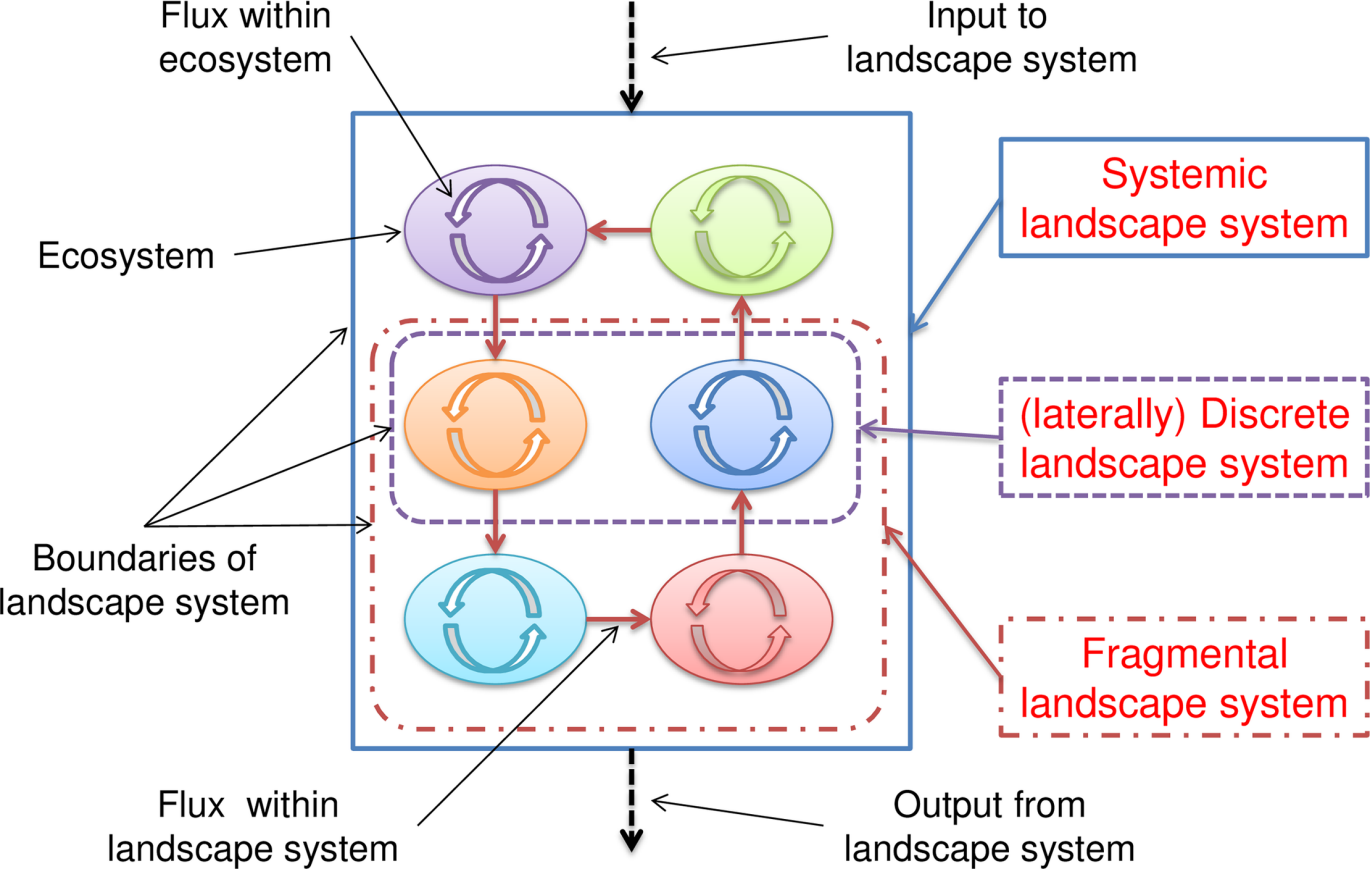
Schematic representation of three types of (three-dimensional) landscape systems represented by a two-dimensional diagram.

Contrasting the features of every type of landscape system with the assumptions of a meta-ecosystem, it is clear that a discrete (either omnidirectionally or laterally) landscape system does not meet the criterion that local ecosystems within the meta-ecosystem should be interconnected; a fragmental landscape system does not meet the criterion that a meta-ecosystem should be closed (or quasi-closed); only a systemic landscape system can be treated as a meta-ecosystem directly (Fig 4).

A fragmental landscape system can be adjusted to a meta-ecosystem by re-delineating its boundary to embrace all ecosystems which have significant fluxes with the ecosystems within the fragmental landscape system. If the fluxes input to/ output from a fragmental landscape system can be simplified to a limited number of exterior compartments and be considered as the components of a closed meta-ecosystem, then the fragmental landscape system can be approximated as a meta-ecosystem too.

In a laterally discrete landscape system, if there are several ecosystems interconnected by vertical fluxes even they are not spatially adjacent, these interconnected ecosystems can compose a new landscape system, which can be treated as a spatially discontinuous meta-ecosystem. An omnidirectionally discrete landscape system would not be adjusted to a meta-ecosystem.

### Integrating landscape system and meta-ecosystem frameworks through meta-ecosystems

“Integrating landscape system and meta-ecosystem frameworks through meta-ecosystems” means that through adjusting landscape systems to meta-ecosystems, an overarching framework is constructed from landscape system framework and meta-ecosystem framework, in which we could study driving factors, landscape heterogeneity, ecosystem processes, ecosystem features and the interactions among them systematically (Fig 1).

Following landscape system framework, researchers could decide how to model the spatial heterogeneity of the meta-ecosystem along the decision tree (Fig 2) [1, 5]. Usually, a meta-ecosystem needs to be analyzed with an interactive model, in which the spatially heterogeneous pattern (both composition and configuration) of a meta-ecosystem could be described, the drivers of the spatially heterogeneous pattern could be discussed, the behavior of a meta-ecosystem processes could be predicted [1, 5, 16, 17].

Following meta-ecosystem framework (Table 1), based on the spatially heterogeneous pattern of a meta-ecosystem, researchers could analyze the local impacts and global constraints of the spatial flows on local ecosystems and the meta-ecosystem, predict the dynamic or evolution of the meta-ecosystem, identify its holistic properties (such as TST, AMI, A, C and R) by using Ecological Network Analysis (ENA) [4, 8, 9].

### Case study

The NHLD is one of the most lake-rich regions of the world, and consists of a mosaic of lakes and wetlands interspersed in a mixed forest landscape. In the landscape, 53% total by area is forests, 28% is wetlands, 13% is lakes and the remainder (5%) includes roads, small towns, agriculture and shrublands [17]. Buffam and others (2011) have estimated the C pools and fluxes for the NHLD region as a whole and for forests, wetlands and lakes respectively [17]. Based on the data set provided by Buffam and others (2011), we analyzed the spatial heterogeneity and ecosystem features of the carbon flows in the NHLD following our protocol.

## Results

### Delineating the NHLD landscape system

**Fig 5:**
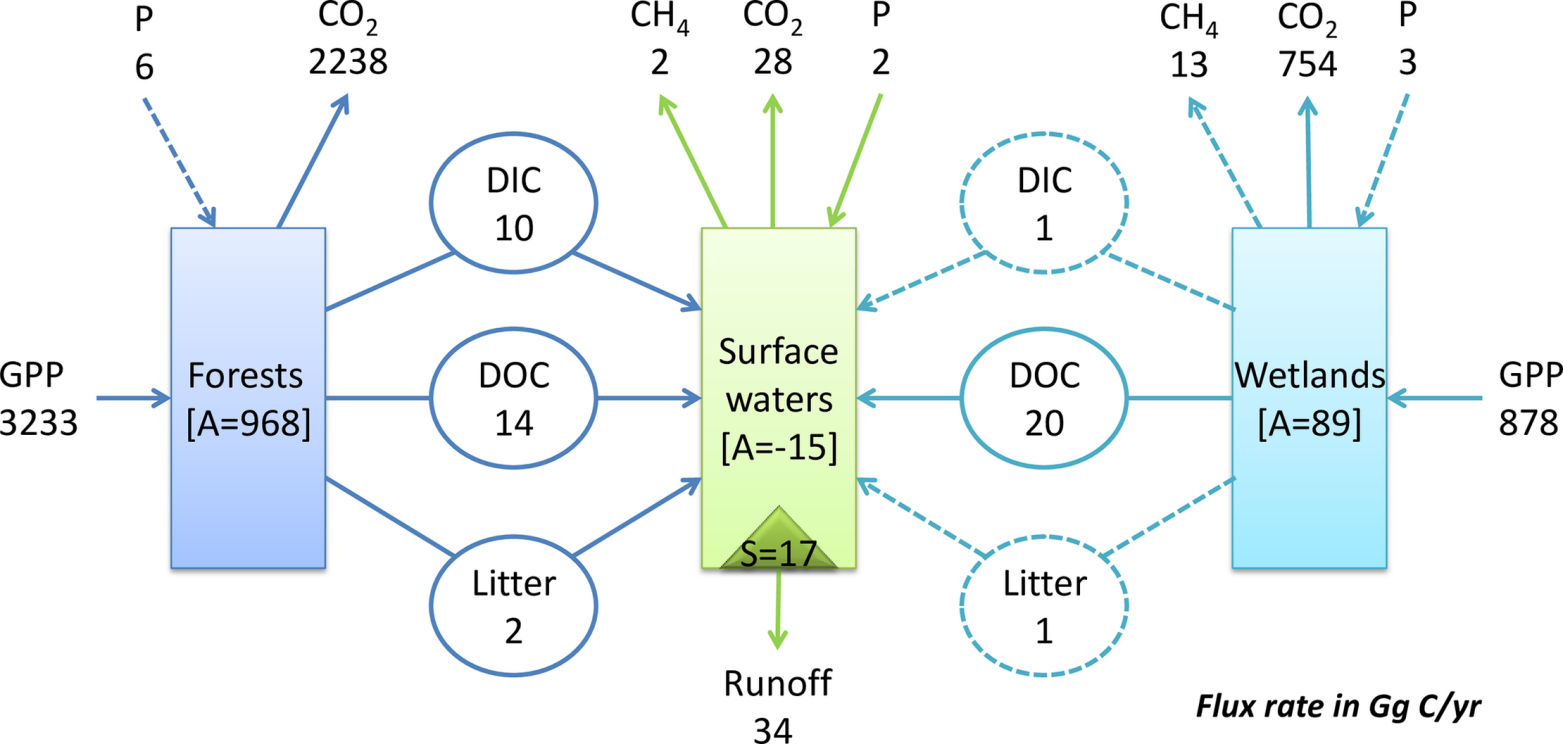
Schematic showing the landscape system of the Northern Highlands Lake District (NHLD), along with best estimates of C flux rates (units of Gg C yr^-1^ for the entire region). These estimates are associated with varying degrees of uncertainty (Table 2). The dashed parts show the fluxes considered insignificant. DOC, dissolved organic carbon; DIC, dissolved inorganic carbon; GPP, gross primary production; P, precipitation; Litter, leaf litter; A, accumulation; S, sedimentation.

Along Buffam and others (2011), we divided the landscape into three major compartments (forests, wetlands and surface waters). Following the protocol, we delineated the NHLD as a landscape system, with each compartment as an individual ecosystem (Fig 5). Then we calculated the major C fluxes into and out of these compartments (Table 2) based on the data sets of C pools and fluxes (please see Table 1–5 provided by Buffam and others (2011)).

**Table 2:** Estimated C fluxes into and out of the three major compartments (forests, wetlands and surface waters) of the NHLD [17].

From	To	Formation	Local Flux, best estimate (range) (g C m^-2^ yr^-1^)	Total Flux, best estimate (range) (Gg C yr^-1^)
External	Forest	GPP	936 (903–969)	3233 (3119–3347)
External	Forest	Precipitation	1.8 (1.2–2.4)	6.2 (4.1–8.3)
Forest	Forest	Accumulation		968 (873–1063)
Forest	External	Respiration (CO_2_)	648 (630–666)	2238 (2176–2301)
Forest	Surface waters	DIC runoff	3.0 (1.3–4.8)	10 (4–17)
Forest	Surface waters	DOC runoff	4.0 (1.5–6.6)	14 (5–23)
Forest	Surface waters	Litter	300 (150–450)^a^	2.3 (1.2–3.5)
External	Wetland	GPP	490 (467–513)	878 (836–919)
External	Wetland	Precipitation	1.8 (1.2–2.4)	3.2 (2.1–4.3)
Wetland	Wetland	Accumulation		89 (27–152)
Wetland	External	Respiration (CO_2_)	421 (379–463)	754 (679–829)
Wetland	External	CH_4_	10 (1–20)	13 (1–25)
Wetland	Surface waters	DIC runoff	0.6 (-1.2–2.3)	1.0 (-2.2–4.2)
Wetland	Surface waters	DOC runoff	11 (2–20)	20 (4–35)
Wetland	Surface waters	Litter	200 (100–300)^b^	0.7 (0.3–1.0)
External	Surface waters	Precipitation	1.8 (1.2–2.4)	1.5 (1.0–2.0)
Surface waters	Surface waters	Accumulation		-15 (-40-9)
Surface waters	Surface waters	Sediment	20 (9–31)	17 (8–26)
Surface waters	External	CO_2_ evasion	33 (26–39)	28 (22–34)
Surface waters	External	CH_4_ evasion	3 (1–4)	2.2 (1.1–3.3)
Surface waters	External	Runoff	5 (4–7)	34 (23–45)

NHLD, Northern Highlands Lake District; GPP, gross primary production; DIC, dissolved inorganic carbon; DOC, dissolved organic carbon.

^a^ Units of g C m^-1^ of shoreline yr^-1^. Leaf litter and other aerial C fluxes from forests to surface waters cross the Forest–Surface water interface (7805km).

^b^ Units of g C m^-1^ of shoreline yr^-1^. Leaf litter and other aerial C fluxes from wetlands to surface waters cross the Wetland–Surface water interface (3469km).

### Classifying the NHLD landscape system

We considered the C fluxes crossing ecosystem boundaries as insignificant if they were two orders of magnitude smaller than the C fluxes within ecosystems (here, we used the TST of every ecosystem). As the TST of C fluxes in the three ecosystems (forests, wetlands and surface waters) respectively were 5503.5 GgC/yr, 1669.9 GgC/yr and 113.7 GgC/yr, (1) the C fluxes of precipitation input to forest and wetland were insignificant, (2) the C fluxes of leaf litter from wetlands to surface waters were insignificant, (3) the C fluxes of wetland DIC runoff to surface waters were insignificant, (4) the C fluxes of wetland CH_4_ emission were insignificant, and (5) the remaining fluxes were significant (Fig 5).

Along the decision tree in the protocol (Fig 3), as (1) GPP and respiration of forests and wetlands vertically crossed the NHLD boundaries, (2) runoff from surface waters to external laterally crossed the NHLD boundaries, (3) DOC runoff from forests and wetlands to surface waters connected adjacent ecosystems of the NHLD, the NHLD was a fragmental landscape system.

### Adjusting the NHLD landscape system to the NHLD meta-ecosystem

**Fig 6:**
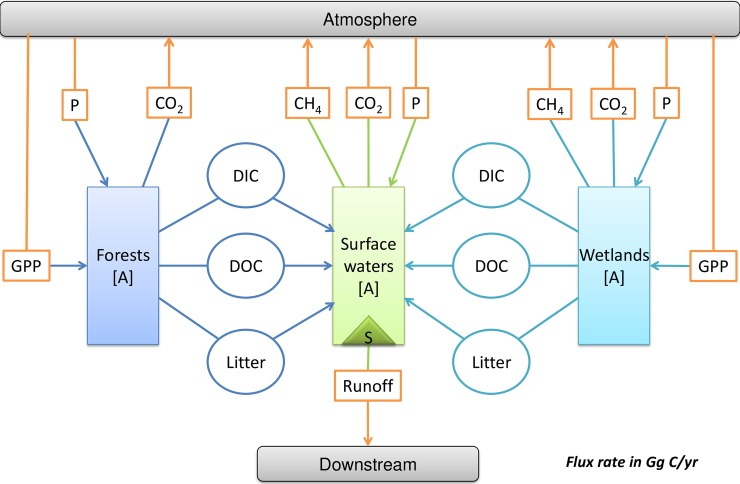
Schematic showing the meta-ecosystem of the Northern Highlands Lake District (NHLD), modified from Fig 5. In the NHLD meta-ecosystem, the atmosphere and downstream are exterior inexhaustible compartments. As all attention was paid on the C fluxes among compartments, atmosphere and downstream would be regarded as the opposite compartments, and its accumulation could be described with negative values. DOC, dissolved organic carbon; DIC, dissolved inorganic carbon; GPP, gross primary production; P, precipitation; Litter, leaf litter; A, accumulation; S, sedimentation.

As the C fluxes crossing NHLD boundaries mainly were vertical (except the runoff from surface waters to external), the fragmental landscape system of NHLD can be adjusted to a meta-ecosystem by simplifying their crossing boundaries fluxes to a limited number of exterior compartments (atmosphere and downstream) (Fig 6).

### Integrating landscape system and meta-ecosystem frameworks through the NHLD meta-ecosystem

To appropriately understand and describe the NHLD meta-ecosystem spatial heterogeneity, we could find out an appropriate method according to the landscape system framework. Following the decision tree (Fig 2), as there were significant lateral C fluxes crossing ecosystems boundaries, such as DOC runoff from forests and wetlands to surface waters, the NHLD meta-ecosystem was best analysed with an interactive model, in which both composition and configuration should be considered.

**Fig 7:**
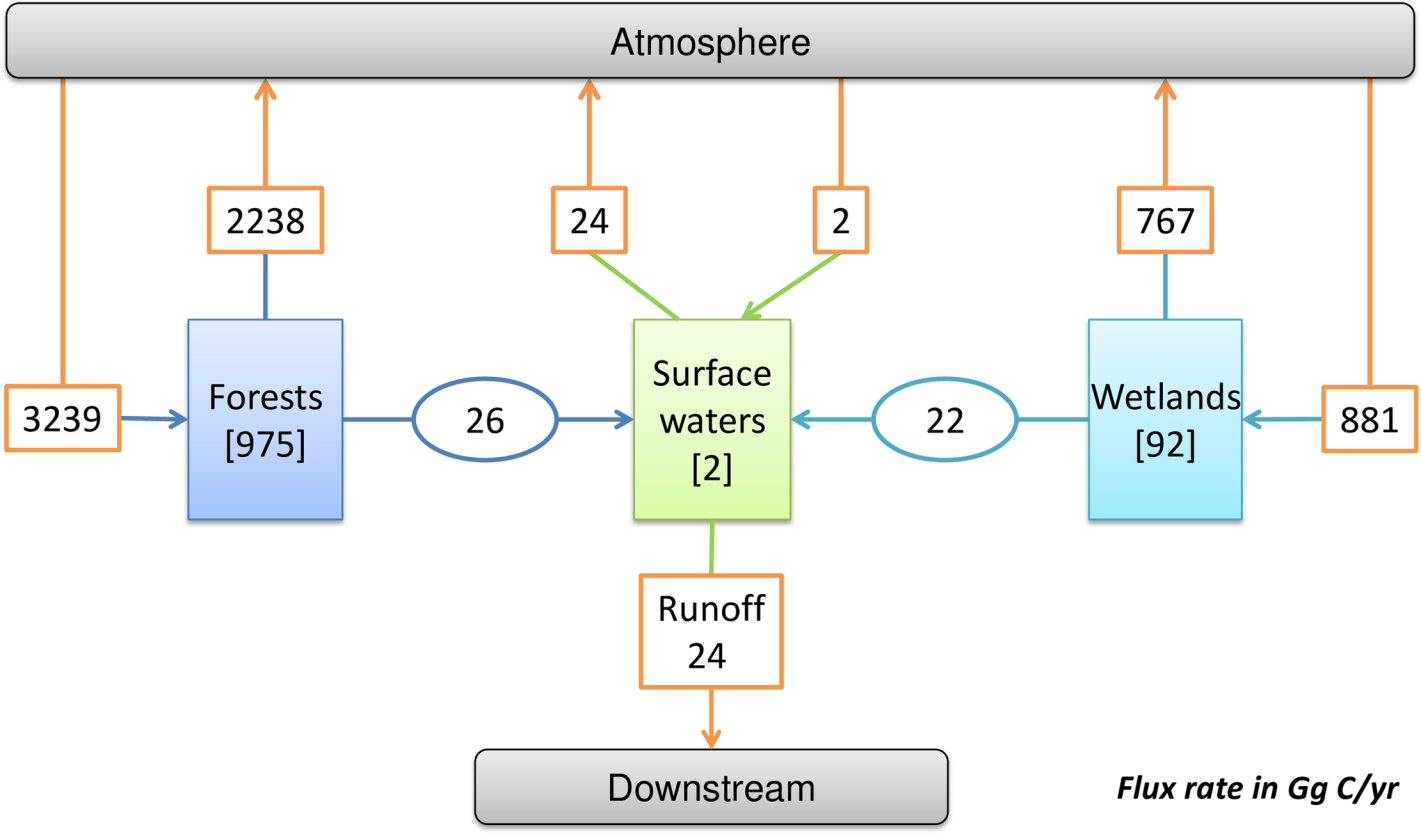
Schematic showing the adjusted C fluxes network (with best estimated fluxes) in the meta-ecosystem of the Northern Highlands Lake District (NHLD), modified from Fig 6. The atmosphere and downstream are exterior inexhaustible compartments.

To identify the holistic features of the NHLD meta-ecosystem, we could use the meta-ecosystem framework. Based on the spatial heterogeneity of the NHLD meta-ecosystem, we constructed the flow network (i.e. the input-output table) of the C fluxes of the NHLD meta-ecosystem (Fig 7), and then calculated its holistic features according to ENA (reference Table 1 explicitly). The results showed that TST, AMI, A, C and R of C fluxes system of the NHLD meta-ecosystem respectively were 8292.00 Gg C yr^-1^, 0.668294 bits, 5541.49 Gg C bits yr^-1^, 11767.45 Gg C bits yr^-1^, 6225.95 Gg C bits yr^-1^.

## Discussion

In the overarching framework constructed by the meta-ecosystem framework and the landscape system framework, one could study the driving factors, landscape heterogeneity, ecosystem processes, ecosystem features and the interactions among them systematically. Here, we analyzed and described the spatial heterogeneity of the NHLD meta-ecosystem, and provided a framework to analyze its driving factors. Then, based on its spatial heterogeneity we analyzed the holistic features of C fluxes system of the NHLD meta-ecosystem.

### Spatial heterogeneity of the NHLD meta-ecosystem

To describe the subtle heterogeneity of the NHLD meta-ecosystem, we could classify the C fluxes of the NHLD into three types: (1) spatially invariable vertical C fluxes, such as the C precipitation; (2) spatially variable vertical C fluxes, such as the GPP and respiration of forests and wetlands, the CH_4_ evasion from wetlands, and the CO_2_ and CH_4_ evasion from surface waters; (3) lateral C fluxes, such as the runoff of DIC and DOC from forests and wetlands to surface waters, the leaf litter from forests and wetlands to surface waters, and the regional riverine runoff from surface waters to downstream. Then, along the decision tree (Fig 2), we could analyze the three types of C fluxes with the homogeneous, mosaic and interactive models, respectively.

The vertical C fluxes within the landscape system of NHLD connect the three main compartments (forests, wetlands, surface waters) with the atmosphere. As being spatially invariable, the mean values of the C precipitation intensity would be sufficient to characterize the C precipitation process within the NHLD meta-ecosystem

$$\frac{F_{f,P}}{A_{f}}=\frac{F_{w,P}}{A_{w}}=\frac{F_{s,P}}{A_{s}}=\frac{F_{all,P}}{A_{all}}=\rho_{P}$$ (1)

In the equation, *F*_*f*,*P*_ denotes the C precipitation input to forests; *A*_*f*_ denotes the area of forests; *ρ*_*P*_ denotes the intensity of C precipitation; the subscript *f*, *w*, *s* and *all* respectively denote forests, wetland, surface waters and all area.

The C fluxes of GPP, respiration and CH4 evasion are spatially variable, and the C fluxes of CO_2_ emission are only estimated in surface waters. Three compartments (forests, wetlands and surface waters) of NHLD could be modeled separately, and then summed the separate results to yield the whole system vertical fluxes.

$$F_{f,v}=\rho_{P}*A_{f}+\rho_{f,GPP}*A_{f}-\rho_{f,R}*A_{f}$$ (2)

$$F_{w,v}=\rho_{P}*A_{w}+\rho_{w,GPP}*A_{w}-\rho_{w,R}*A_{w}-\rho_{w,CH4}*A_{w}$$ (3)

$$F_{s,v}=\rho_{P}*A_{s}-\rho_{s,CO2}*A_{s}-\rho_{s,CH4}*A_{s}$$ (4)

$$F_{a,v}=-(F_{f,v}+F_{w,v}+F_{s,v})$$ (5)

In these equations, *F*_*a*,*v*_ denotes the vertical C fluxes input to atmosphere, *ρ*_*f*,*GPP*_ denotes the intensity of forest GPP; *ρ*_*f*,*R*_ denotes the intensity of forest respiration; *ρ*_*s*,*CO2*_ and *ρ*_*s*,*CH4*_ respectively denote the intensity of CO_2_ and CH_4_ evasion from surface waters.

The lateral C fluxes within the NHLD meta-ecosystem connect the four lateral compartments (forests, wetlands, surface waters and downstream). The compositions and the configurations of the NHLD meta-ecosystem should be considered simultaneously (Fig 6).

$$F_{f,l}=-(\rho_{f,l,DIC}*A_{f}+\rho_{f,l,DOC}*A_{f}+\rho_{f,l,litter}*L_{f})$$ (6)

$$F_{w,l}=-(\rho_{w,l,DIC}*A_{w}+\rho_{w,l,DOC}*A_{w}+\rho_{w,l,litter}*L_{w})$$ (7)

$$F_{s,l}=\rho_{f,l,DIC}*A_{f}+\rho_{f,l,DOC}*A_{f}+\rho_{f,l,litter}*L_{f}+\rho_{w,l,DIC}*A_{w}+\rho_{w,l,DOC}*A_{w}+\rho_{w,l,litter}*L_{w}-F_{d,l}$$ (8)

In these equations, *ρ*_*f*,*l*,*DIC*_ denotes the intensity of the DIC runoff fluxes from forests to surface waters; *ρ*_*w*,*l*,*DIC*_ denotes the intensity of the DIC runoff fluxes from wetlands to surface waters; *L*_*f*_ denotes the length of forests-surface waters.

In the NHLD, there were spatially invariable vertical fluxes, spatially variable vertical fluxes and lateral fluxes simultaneously. The spatially variable vertical fluxes determined the necessity for considering the compositions of the NHLD meta-ecosystem. And then the lateral fluxes determined the necessity for considering the configuration of the NHLD meta-ecosystem. That is, the spatial heterogeneity of NHLD meta-ecosystem should be described with a triple system.

Of course, based on the spatial heterogeneity of the NHLD meta-ecosystem, we could also discuss the drivers of these heterogeneous processes, such as the drivers of the C precipitation fluxes, the drivers of the C fluxes of GPP, respiration, CH_4_ evasion, CO_2_ emission and sediment deposition, the drivers of the C fluxes of DIC and DOC runoff and leaf litter subsidy, and so on [17–20].

### Holistic features of C fluxes system of the NHLD meta-ecosystem

The indicators in ENA provide unified benchmarks for holistic functional assessment of meta-ecosystems [9, 21]. In the NHLD meta-ecosystem, the C fluxes system size (TST) is 8292.00 Gg C yr^-1^. Comparing with the C pool size (380.05 Tg C) [17], the C fluxes system is large enough (2.18%), which means it is very active. The C fluxes system average uncertainty (AMI) is 0.668294 bits, which shows that to identify the direction of C flux out of a compartment, one need to make binary decision 0.668294 times only. In another word, the connectivity among compartments is high and the system organization is low in this C fluxes system. As the A, C and R respectively are 5541.49 Gg C bits yr^-1^, 11767.45 Gg C bits yr^-1^, 6225.95 Gg C bits yr^-1^, the resilience of C fluxes system is relatively high (52.91%) in the NHLD meta-ecosystem.

**Fig 8:**
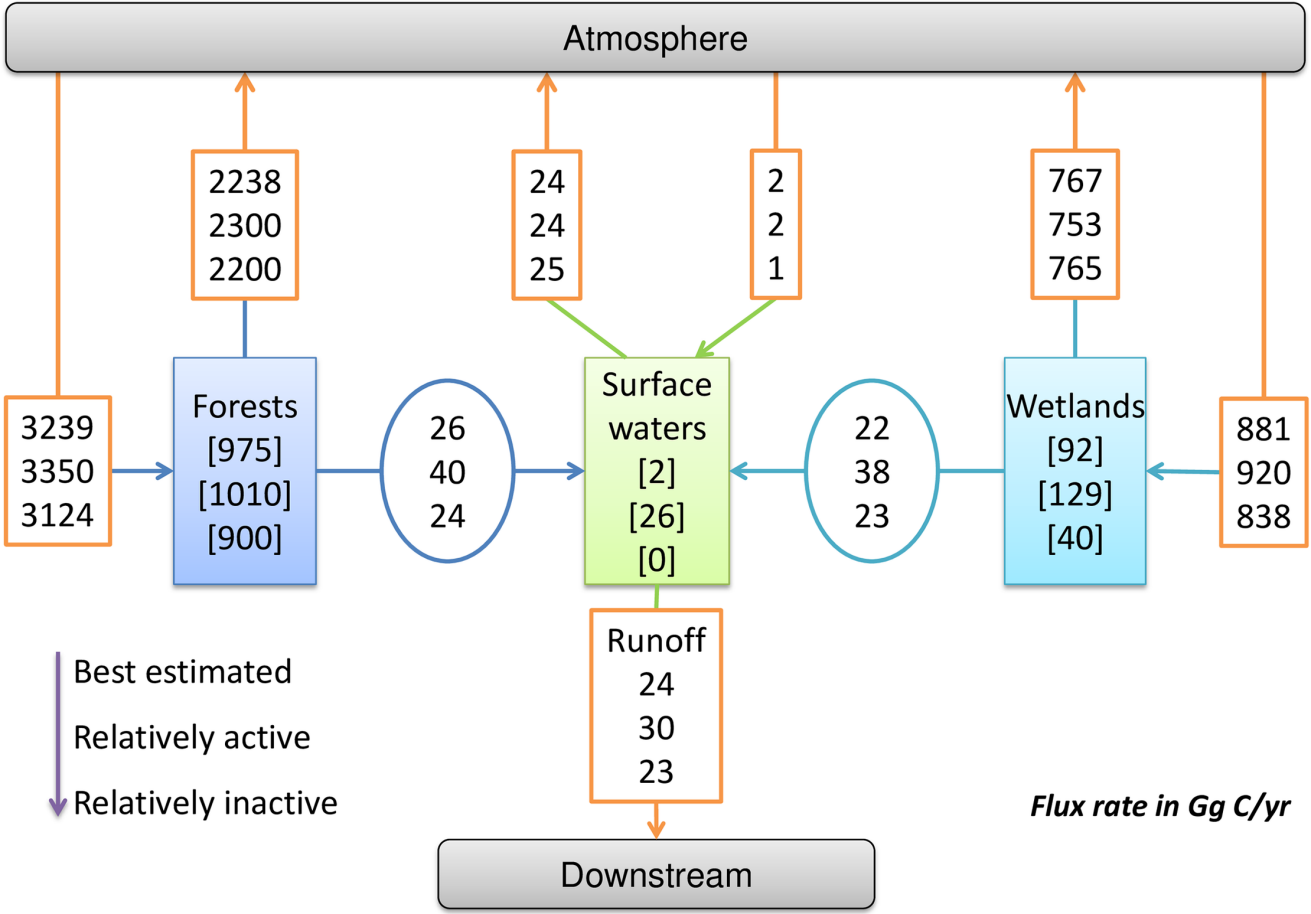
Schematic showing three C fluxes network (i.e. the upper for the best estimated one, the middle for the relatively active one and the lower for the relatively inactive one) in the meta-ecosystem of the Northern Highlands Lake District (NHLD), following Fig 7. In the C fluxes network of “best estimated” scenario, we used the C fluxes with best estimated net accumulation and fluxes. In the C fluxes network of “relatively active” scenario, we used a set of C fluxes with relatively higher net accumulation and fluxes. In the C fluxes network of “relatively inactive” scenario, we used a set of C fluxes with relatively lower net accumulation and fluxes. In this C fluxes network, the atmosphere and downstream are exterior compartments.

For discussing the variation of activity, organization and resilience of C fluxes system in the NHLD meta-ecosystem, we calculated its TST, AMI, A, C and R in three scenarios (i.e. best estimated, relatively active and relatively inactive) (Fig 8).

The results of ENA (Table 3) showed that TST, A, C and R of the C fluxes network in the relatively active scenario were significantly larger than the ones in the best estimated scenario, and than the ones in the relatively inactive scenario. It meant that there was a propensity that the meta-ecosystem of NHLD would becoming more active, more developed, more robust if the C fluxes were relatively higher.

**Table 3:** TST, AMI, A, C and R of the C fluxes network in three scenarios (i.e. best estimated, relatively active and relatively inactive) of the Northern Highlands Lake District (NHLD) meta-ecosystem.

Holistic features	Best estimated	Relatively active	Relatively inactive
TST (Gg C yr^-1^)	8292.00	8622.00	7973.00
AMI (bits)	0.668294	0.675669	0.668200
A (Gg C bits yr^-1^)	5541.49	5825.62	5327.56
C (Gg C bits yr^-1^)	11767.45	12407.15	11297.23
R (Gg C bits yr^-1^)	6225.95	6581.53	5969.67

### The overarching framework for completely understanding heterogeneous ecological systems

As the integration of landscape system and meta-ecosystem frameworks constructing an overarching framework for understanding heterogeneous ecological systems, all topics about heterogeneous landscapes could be studied in this overarching framework. For example, following our protocol, one could construct a meta-ecosystem at any study area and spatial scale, and then (1) choose an appropriate model to analyse and describe its spatial heterogeneity [5], (2) analyse and predict the behavior of each spatially heterogeneous flux based on the spatially heterogeneous pattern and their corresponding drivers [22], (3) analyze and predict the behavior of local ecosystems and meta-ecosystem based on local impacts and global constraints of fluxes [11, 23], (4) analyze and evaluate the state of the meta-ecosystem through the unified benchmarks for holistic functional assessment of meta-ecosystems using ENA [9, 24]. We agree that most of these works could be done in other frameworks. But we believe that doing them in our framework would be better, because our framework provides an overarching framework.

Moreover, we promise that our protocol would benefit the development of some ecological theories. For example, after the seminal paper that proposed the concept of meta-ecosystem and the idea of global constraints (meta-ecosystem theory), there have been many works to extend this concept and idea into spatial ecosystem ecology [25–27], which are valuable to understanding the ecosystem processes in heterogeneous ecological systems. However, to date, meta-ecosystem theory and associated studies mainly focused on theoretical analysis [27–33], and few empirical studies have been conducted [34–36]. It is suggested that one of the barriers is that it is difficult to identify a closed meta-ecosystem with mass conservation because the set of interactive ecosystems is open to inputs and outputs, and therefore, internal sources and sinks cannot be in balance [5]. Here, our protocol provides a general method to identify a (*quasi-*) closed meta-ecosystem. The construction of empirical meta-ecosystems will advance the empirical development of meta-ecosystem theory, particularly shed light on the empirical studies of spatial heterogeneous ecological system evolution.

## Conclusions

In this study, we propose a protocol of integrating meta-ecosystem framework with landscape system framework. The integration constructs an overarching framework, in which the studies on driving factors, landscape heterogeneity, ecosystem processes, ecosystem features and the interactions among them of a heterogeneous ecological system are covered thoroughly (Fig 1). As the subsidies of nutrients and energy among ecosystems are always important for ecosystem sustainability, to understand and determine the patterns, causes and effects of a heterogeneous ecological system is a key topic in ecology. Although there were many works [1], there was no overall conceptual framework. This overarching framework would be conducive to completely understanding a heterogeneous ecological system.

Practically, following our protocol one could construct a meta-ecosystem and analyze it in the overarching framework. Such as in this contribution, we constructed a meta-ecosystem based on the C fluxes in the NHLD, and then analyzed its spatial heterogeneity and holistic features. Furthermore, one could also construct a watershed as a meta-ecosystem based on the nutrients and water flows, in which there would be several types’ ecosystems, such as forest, farm, grassland, wetland, river and lake, and then analyze its spatial heterogeneity, driving factors, ecological processes, ecosystem features and evolution.
